# Validation of a cervical CDO1/CELF4 methylation test for endometrial cancer: a prospective paired-sample comparison with intrauterine specimen

**DOI:** 10.3389/fmed.2026.1690020

**Published:** 2026-05-29

**Authors:** Bingxin Cai, Yishan Wang, Wen Ma, Zhenzhen Wu, Tingting Yao, Pei Liu, Linhai Wang, Pingping Zhou, Yilin Li

**Affiliations:** 1 Department of Obstetrics and Gynecology, Gansu Provincial Maternity and Child-care Hospital, Lanzhou, China; 2 Department of Medical Laboratory, Beijing Origin-Poly Bio-Tec Co., Ltd., Beijing, China; 3 Law School of Chengdu University, Chengdu, China

**Keywords:** biomarkers, cervical brushing cells, DNA methylation, early detection of cancer, endometrial neoplasms

## Abstract

**Background:**

The increasing global incidence of endometrial cancer (EC) underscores the urgent need for effective early detection. Noninvasive detection approaches, such as transvaginal ultrasonography (TVS), frequently exhibit low specificity, leading to a high rate of unnecessary invasive procedures. DNA methylation analysis has emerged as a promising noninvasive alternative. This study aimed to validate *CDO1/CELF4* gene methylation analysis for detecting endometrial intraepithelial neoplasia (EIN) and EC and, critically, to compare its diagnostic efficacy using noninvasive cervical brushing cells (CBCs) vs. invasive diagnostic curettage (D&C) samples.

**Methods:**

A prospective paired-samples study was conducted at Gansu Provincial Maternity and Child Care Hospital (July 2023–June 2024), including 226 eligible women. CBC and D&C samples were collected sequentially from each participant and tested for CDO1/CELF4 methylation, with diagnostic performance and consistency compared between sample types.

**Results:**

Among 226 participants, 31 were diagnosed with EIN or EC. Compared with conventional markers (cytology, endometrial thickness, and CA125 levels), individual *CDO1*, *CELF4*, and CISENDO (*CDO1/CELF4*) methylation tests from both CBC and D&C samples demonstrated superior diagnostic capabilities. Specifically, the ΔCt values of *CDO1* and *CELF4* were significantly lower in patients with EIN or EC than in those with benign lesions (*P* < 0.0001 for all comparisons). A strong positive correlation was observed between CBC and D&C samples for individual methylated *CDO1* tests (*R* = 0.86) and *CELF4* tests (*R* = 0.78), suggesting a high agreement. ROC analysis confirmed no statistically significant difference in diagnostic accuracy between CBC and D&C samples for the CISENDO test (*P* = 0.244). The CISENDO test achieved an AUC of 0.929, with 93.5% sensitivity and 92.3% specificity using D&C samples, and an AUC of 0.916 with CBC samples when using manufacturer-recommended cutoff.

**Conclusion:**

*CDO1/CELF4* methylation analysis in cervical brushing samples is a highly accurate and reliable method for detecting EC and precursors, and no statistically significant difference in efficacy was observed compared to that of endometrial tissue tests. Its noninvasive nature can overcome significant diagnostic barriers, holding considerable promise as an effective triage tool for high-risk or symptomatic women, potentially revolutionizing early detection of EC and improving patient outcomes.

## Background

Over the past three decades, the global incidence rate of endometrial cancer (EC) has increased by 132% (1), with 420,368 new cases reported in 2022 alone, driven by factors such as obesity and an aging population (2). Notably, there has been a substantial increase in the number of EC cases among women under 40 years of age, who now constitute 4.2% of all low-grade ECs diagnosed in the United States (3), highlighting the growing incidence of EC among younger individuals. Notably, the incidence rate of EC has risen more sharply in high-income regions than in low-income areas. Conversely, economic factors contribute to a markedly elevated mortality rate from EC among patients residing in low- and middle-income regions (4). Despite a 15% reduction in EC mortality rates in recent years, the absolute number of EC-related deaths has nevertheless risen (1).

The need for precise early detection of EC is critical, as timely diagnosis markedly enhances patient outcomes. For patients with stage I EC, the 5-year survival rate can reach up to 90%, whereas it can reach 50–60% for stage III EC and fall below 20% for stage IV EC (5, 6). Effective early detection of EC not only improves patient prognosis but also mitigates the need for extensive surgery or adjuvant treatments, thereby reducing treatment costs and minimizing morbidity and mortality (7). Currently, abnormal uterine bleeding (AUB), especially postmenopausal bleeding (PMB), is the typical symptom reported by more than 90% of EC patients (8). While early diagnosis relies heavily on invasive procedures such as dilation and curettage (D&C), hysteroscopy, and subsequent pathological examination of endometrial tissues (9), these methods are associated with patient discomfort, pain, and poor compliance (10). Conversely, non-invasive clinical approaches, such as evaluating body mass index (BMI), measuring endometrial thickness (ET) via transvaginal ultrasonography (TVS), and assessing serum carbohydrate antigen 125 (CA125) levels, have demonstrated detection limitations (11, 12). Although TVS has high sensitivity for EC detection, its low specificity frequently leads to unnecessary invasive procedures (13). Critically, no universally recommended noninvasive EC screening protocols currently exist for clinical practice (14).

To address these diagnostic gaps, DNA methylation detection has emerged as a promising and effective noninvasive approach for cancer screening and triage (7). Aberrant DNA methylation, particularly in the promoter regions of tumor suppressor genes, can lead to gene silencing and contribute to cancer progression (15). Recent research has focused on the potential of such epigenetic biomarkers for early EC detection via minimally invasive samples (7). Among these methods, the *CDO1* (cysteine dioxygenase type 1) and *CELF4* (CUGBP Elav-like family member 4) gene methylation tests have been identified as highly effective methods for identifying endometrial intraepithelial neoplasia (EIN) and EC lesions. For example, the dual-gene (*CDO1*/*CELF4*) methylation (CISENDO^®^) test using cervical brushing cells has exhibited high sensitivity (87.5%) and specificity (95.9%) in postmenopausal women with suspected endometrial lesions (16) and comparable results (85.7% sensitivity and 87.6% specificity) in premenopausal women with AUB (17). This method has also demonstrated the ability to distinguish between benign and malignant tumors and can further improve screening sensitivity when combined with TVS (16–18). While the clinical superiority of the CISENDO^®^ test using cervical brushing cells for identifying endometrial lesions has been validated, a critical clinical question regarding the comparison between sample types—namely, cervical brushing cells vs. endometrial scraping cells or tissue—remains unresolved. Specifically, concerns persist about whether CBC, which is noninvasive and convenient to collect, can achieve comparable accuracy to that of endometrial scraping cells/tissues obtained through more invasive procedures, such as D&C or hysteroscopy.

Therefore, the primary objectives of this study are twofold: first, to provide further evidence for the performance of the CISENDO^®^ test in detecting EIN and EC based on CBC and D&C specimens; second, and more critically, to directly compare the diagnostic efficacy of the CISENDO^®^ test using two different sample types: cervical brushing and D&C collection. We aimed to validate the clinical utility and feasibility of using the CBC to reliably compare samples obtained by D&C for the noninvasive triage of women at increased risk for EC, particularly those with clinical indications warranting further diagnostic evaluation. This would not only increase patient comfort and accessibility but also potentially allow for integration with existing cervical cancer screening programs, potentially transforming the early detection landscape for EC.

## Materials and methods

### Study design, participants, and specimen collection

**FIGURE 1:**
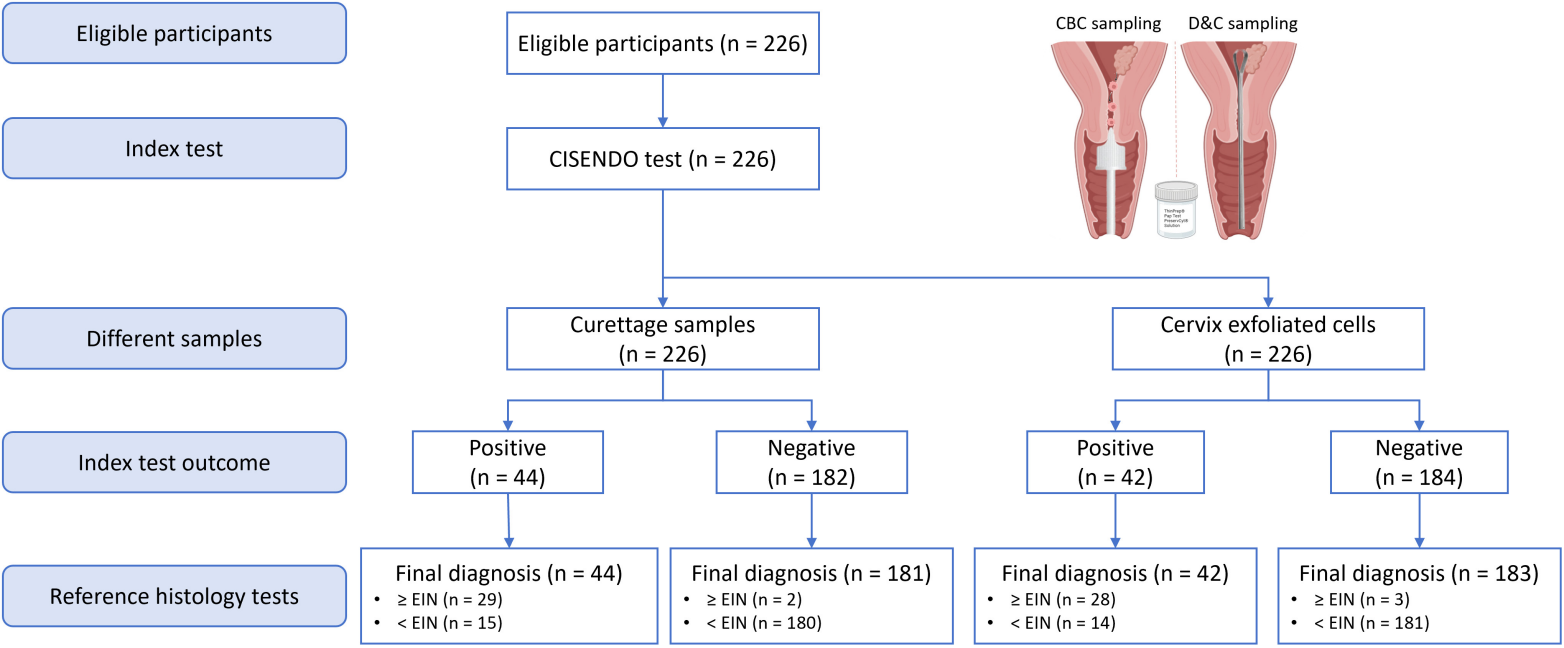
Study flow diagram adhering to the STARD guidelines. Study design and participant flow diagram. Participant recruitment, sample collection, and diagnostic procedures adhered to the Standards for Reporting Diagnostic Accuracy Studies (STARD) guidelines. A total of 226 eligible participants underwent the CISENDO methylation test. Samples were collected via two methods: diagnostic curettage (D&C) and noninvasive cervical brushing (CBC). For D&C samples: 44 tested positive for CISENDO, of which 29 were histologically confirmed as ≥ endometrial intraepithelial neoplasia (EIN) and 15 as < EIN; 182 tested negative, with 2 confirmed as ≥ EIN and 180 as < EIN. For CBC samples: 42 tested positive, with 28 confirmed as ≥ EIN and 14 as < EIN; 184 tested negative, with 3 confirmed as ≥ EIN and 181 as < EIN. All final diagnoses were confirmed by reference histology.

This prospective, paired study was conducted from July 2023 to June 2024 and included 226 eligible women from the gynecology outpatient clinic at Gansu Provincial Maternity and Child Care Hospital, Gansu, China (Figure 1). The study was approved by the Ethics Committee of Gansu Provincial Maternity and Child Health Care Hospital (Ethics Approval Number: IRB No. 2023-GSFY-56). Participants were included if they were over 18 years of age, had an indication for hysteroscopy referral or uterine cavity surgery, and provided signed informed consent. Women who were pregnant, those with a history of hysterectomy, and those with incomplete case data were excluded from the study. All participants reviewed and signed informed consent in accordance with the Standards for Reporting of Diagnostic Accuracy Studies (STARD) (19).

Following signed informed consent, during the same clinical visit, the physician consistently collected CBCs first via a Cervex Brush (Rovers Medical Devices, Netherlands) to avoid contamination with endometrial tissue. Immediately following this, the D&C specimen was subsequently preserved after hysteroscopy-guided biopsy or D&C. All collected samples were immediately preserved in PreservCyt^®^ solution (Hologic, Bedford, MA, United States), stored at 4°C, and subjected to a CISENDO^®^ test within one week after collection. The pathological results were obtained and validated by two specialized gynecologic pathologists. Clinical information, including age, presenting symptoms, BMI, CA125 test results, and ET measured via TVS, was systematically collected.

### Detection procedure for the CDO1 and CELF4 methylation (CISENDO^®^) test

Methylation analysis was performed in a certified DNA detection laboratory in Beijing Origin-Poly Bio-Tec Co., Ltd, where operators were blinded to the clinical data. Genomic DNA (gDNA) was isolated from CBC and D&C samples via a JH-DNA Isolation and Purification Kit (OriginPoly Bio-Tec Co., Ltd., Beijing, China) following the manufacturer’s protocol. The DNA concentration was determined with a NanoDrop 2000c spectrophotometer (Thermo Fisher Scientific, DE, United States). Subsequently, 500 ng of gDNA was bisulfite-converted via the JH-DNA Methylation-Lightning MagPrep Kit (OriginPoly Bio-Tec Co., Ltd., Beijing, China). The methylation status of *CDO1* and *CELF4* was evaluated via the CISENDO DNA Methylation Detection Kit for Endometrial Cancer (real-time PCR) (OriginPoly Bio-Tec Co., Ltd., Beijing, China; Class III Medical Device Registration Certificate of China: No. 20243402610), with glyceraldehyde-3-phosphate dehydrogenase (*GAPDH*) as an internal reference on the SLAN-96P Real-Time PCR System platform (Shanghai Hongshi Medical Technology Co., Ltd., Shanghai, China). Elevated methylation levels of the *CDO1*/*CELF4* genes were determined by differential ΔCt values (ΔCtC1 = Ct*_*CDO1*_* - Ct*_*GAPDH*_* and ΔCtC4 = Ct*_*CELF4*_* - Ct*_*GAPDH*_*), with lower ΔCt values indicating higher gene methylation levels. A positive outcome for the CISENDO methylation test was established as ΔCtC1 ≤ 8.4 or ΔCtC4 ≤ 8.8, as stipulated by the manufacturer-provided and National Medical Products Administration (NMPA) validated cutoffs. These diagnostic thresholds were uniformly applied to all samples (both CBC and D&C) across all patient subgroups in this study.

### Statistical analysis

In this study, for primary clinical evaluation, established clinical thresholds were used, positive TVS findings were defined as an ET ≥ 11 mm in premenopausal women and an ET ≥ 5 mm in postmenopausal women, and CA125 levels ≥ 35 U/ml were defined as abnormal based on established clinical guidelines. Statistical analysis was performed via R version 4.4.3. In the main text, categorical data are presented as counts and percentages, and group comparisons were conducted via the chi-square test. Continuous data, which did not strictly conform to a normal distribution in this study, are presented as medians and interquartile ranges, and comparisons were performed via the Wilcoxon test. Spearman’s rank correlation coefficient was used to assess the monotonic association between the results obtained from the two sampling methods. ΔCt values were log-transformed prior to agreement and correlation analyses to stabilize variance and approximate normality. All ΔCt values were strictly positive; therefore, logarithmic transformation was mathematically valid without addition of a constant. The Bland–Altman method was used to evaluate sample consistency between the two sampling methods. To test whether the mean difference (bias) between the two sampling methods significantly deviated from zero, a paired t-test was performed alongside the Bland–Altman analysis. Receiver operating characteristic (ROC) curves were plotted to evaluate the accuracy of each indicator in detection for EC, and the area under the curve (AUC) with its 95% confidence interval (95% CI), sensitivity, and specificity were calculated. Additionally, for exploratory purposes, optimal data-driven thresholds for TVS and CA125 were derived using the Youden index from ROC curve analysis to evaluate their maximum potential diagnostic performance against the methylation markers. For methylation markers, both manufacturer-recommended cutoffs and data-driven optimal cutoffs were evaluated. The latter were derived from the current dataset using ROC curve analysis and the Youden index and are therefore considered exploratory. No external validation cohort was available. To reduce optimism bias, internal validation was performed using bootstrap resampling (1,000 iterations) to estimate corrected AUC values. These findings require external validation before clinical implementation. The Delong test was used to compare differences in the diagnostic AUC between different diagnostic strategies. A two-tailed *p*-value of less than 0.05 was considered to indicate statistical significance. All missing data were excluded from the statistical analysis of the relevant variables.

## Results

### Baseline characteristics of the participants

A total of 226 participants were included in the study, of whom 195 had benign lesions and 31 had EIN (*n* = 9) or EC (*n* = 22). Patients with ≥ EIN were older than those with < EIN were (median 49 vs. 42 years; *p* = 0.003), and the age difference was statistically significant when comparing EC patients to non-EC patients (median 56 vs. 42 years; *p* = 0.001). BMI was similar across all comparisons, with no significant difference between groups (*P* = 0.4 and *P* = 0.20, respectively) (Table 1 and Supplementary Table 1).

**TABLE 1:** Baseline characteristics of the patients.

	Pathology		
Characteristic	<EIN *N* = 195^1^	≥ EIN *N* = 31^1^	*p*-value
Age	42 (38, 49)	49 (38, 60)	**0.003^2^**
BMI	23.62 (21.78, 25.71)	24.44 (21.99, 26.84)	0.4^2^
Menopausal			**< 0.001^3^**
Premenopausal	156 (80.00%)	16 (51.61%)	
Postmenopausal	39 (20.00%)	15 (48.39%)	
Symptoms and diseases			0.2^4^
Abnormal bleeding	146 (74.87%)	21 (67.74%)	
Menstrual disorder	31 (15.90%)	4 (12.90%)	
Abdominal pain and distension	7 (3.59%)	1 (3.23%)	
Intrauterine effusion	3 (1.54%)	1 (3.23%)	
Endometrial hyperplasia	8 (4.10%)	4 (12.90%)	

^1^Median (Q1, Q3); n (%);

^2^Wilcoxon rank sum test;

^3^Pearson’s Chi-squared test;

^4^Fisher’s exact test. EIN, endometrial intraepithelial neoplasia; BMI, body mass index. The numbers shown in bold indicate a *p*-value less than 0.05.

Menopausal status was significantly associated with pathological severity. Among patients with ≥ EIN, 48.39% were postmenopausal, whereas 20.00% of those with < EIN were postmenopausal (*P* < 0.001). This difference was more striking in the EC group, where approximately 60% were postmenopausal, vs. 20.1% in the non-EC group (*P* < 0.001).

Symptom patterns also varied with pathology. Abnormal bleeding was the most common symptom across all groups and was less frequently reported in patients with ≥ EIN (67.74% vs. 74.87%) and EC (72.73% vs. 74.02%); however, these differences were not statistically significant (*p* = 0.2 and *p* = 0.8, respectively). Other symptoms, such as menstrual disorders, abdominal pain and distension, were also less prevalent in the ≥ EIN group, yet these trends were not statistically significant.

### Performance of different diagnosis methods

The diagnostic performance of various methods was evaluated in distinguishing EIN and EC (EIN/EC) patients from non-EIN/EC patients stratified by menopausal status (Table 2 and Supplementary Table 2). Furthermore, the characteristics of patients were examined across different pathological categories on the basis of the CISENDO results (Supplementary Table 3).

**TABLE 2:** Diagnostic method performance between patients with < EIN and those with ≥ EIN.

	Premenopausal			Postmenopausal		
Characteristic	< EIN *N* = 156^1^	≥ EIN *N* = 16^1^	*p*-value	< EIN *N* = 39^1^	≥ EIN *N* = 15^1^	*p*-value
**Cytology**			0.3^2^			0.6^2^
NILM	147 (94.23%)	14 (87.50%)		36 (92.31%)	13 (86.67%)	
≥ ASC-US	9 (5.77%)	2 (12.50%)		3 (7.69%)	2 (13.33%)	
Endometrium thickness	10.00 (8.00, 13.00)	10.50 (6.30, 12.50)	0.8^3^	4.00 (2.00, 7.00)	9.00 (4.00, 10.50)	0.018^3^
**ET**			0.8^4^			0.10^4^
< 11 mm (pre)/< 5 mm (post)	83 (53.21%)	8 (50.00%)		20 (51.28%)	4 (26.67%)	
≥ 11 mm (pre)/≥ 5 mm (post)	73 (46.79%)	8 (50.00%)		19 (48.72%)	11 (73.33%)	
CA125( ≥ 35 U/mL)			0.6^2^			0.3^2^
Negative	144 (92.31%)	16 (100.00%)		37 (100.00%)	14 (93.33%)	
Positive	12 (7.69%)	/		/	1 (6.67%)	
Methylated CDO1(CBC)	16.27 (12.95, 17.93)	3.41 (2.71, 5.11)	< 0.001^3^	15.33 (11.76, 17.79)	4.70 (2.32, 6.00)	< 0.001^3^
Methylated CELF4(CBC)	16.94 (13.75, 18.43)	5.05 (2.70, 10.41)	< 0.001^3^	16.98 (12.06, 17.91)	4.65 (3.12, 7.04)	< 0.001^3^
CISENDO(CBC)			< 0.001^2^			<0.001^4^
Negative	148 (94.87%)	1 (6.25%)		33 (84.62%)	2 (13.33%)	
Positive	8 (5.13%)	15 (93.75%)		6 (15.38%)	13 (86.67%)	
Methylated CDO1(D&C)	13.25 (11.19, 15.22)	3.49 (2.97, 5.15)	< 0.001^3^	13.59 (10.63, 15.52)	4.77 (2.39, 5.11)	< 0.001^3^
Methylated CELF4(D&C)	13.31 (11.39, 15.43)	5.00 (2.77, 10.50)	< 0.001^3^	13.67 (11.80, 16.80)	4.85 (1.99, 6.31)	< 0.001^3^
CISENDO(D&C)			< 0.001^2^			<0.001^4^
Negative	145 (92.95%)	1 (6.25%)		35 (89.74%)	1 (6.67%)	
Positive	11 (7.05%)	15 (93.75%)		4 (10.26%)	14 (93.33%)	

^1^n (%); Median (Q1, Q3);

^2^Fisher’s exact test;

^3^Wilcoxon rank sum test;

^4^Pearson’s Chi-squared test. EIN, endometrial intraepithelial neoplasia; NILM, Negative for Intraepithelial Lesion or Malignancy; ASC-US, Atypical Squamous Cells of Undetermined Significance; ET, Endometrium Thickness; CA125, Carbohydrate Antigen 125; CBC, cervical brushing cell; D&C, diagnostic curettage.

Across both premenopausal and postmenopausal cohorts, conventional markers such as cytological findings [Negative for Intraepithelial Lesion or Malignancy (NILM) or Atypical Squamous Cells of Undetermined Significance (ASC-US)], ET (according to classic thresholds), and CA125 levels did not significantly differentiate EIN or EC patients from non-EIN/EC patients (Table 2 and Supplementary Table 2). However, the EC group had a thicker endometrium than the non-EC group did in the postmenopausal cohort.

In contrast, a consistent pattern emerged with specific molecular markers. For both premenopausal and postmenopausal women, the presence of EIN or EC was associated with significantly lower *CDO1* (CBC), *CELF4* (CBC), *CDO1* (D&C), and *CELF4* (D&C) ΔCt values than non-EIN/EC controls (Table 2 and Supplementary Table 2). Moreover, a significantly greater proportion of positive results for CISENDO (CBC) and CISENDO (D&C) was observed in both the EIN and EC patient groups, irrespective of menopausal status (Table 2 and Supplementary Table 2).

Further analysis revealed distinct pathological associations with CISENDO positivity (Supplementary Table 3). Positive CISENDO (both CBC and D&C) results were predominantly observed in patients with EIN and various forms of EC, including endometrioid, serous, and clear cell types. Conversely, patients with benign uterine pathology, polyps, or fibroids predominantly yielded negative CISENDO results, although a minority presented with positive findings. The minority of benign cases presenting with positive CISENDO findings may represent early molecular alterations or field cancerization effects that precede visible morphological changes, or underlying age-related methylation drift, warranting closer longitudinal follow-up for these patients. Notably, 2/3 of type II ECs were positively detected by CISENDO (CBC), and all of them were detected by CISENDO on the basis of the D&C sample, indicating that this methylation panel performs robustly across different pathological subtypes, including the more aggressive type II lesions.

These findings collectively suggest that while traditional markers may lack discriminatory power, *CDO1*, *CELF4*, and the CISENDO test hold promise as potential diagnostic indicators for both EIN and EC. The consistent trends observed across diverse patient populations and menopausal categories, coupled with the robust pathological correlation of the CISENDO with premalignant and malignant uterine conditions, underscore its potential clinical utility.

### Distribution of ΔCt values for methylated *CDO1/CELF4* genes

**FIGURE 2:**
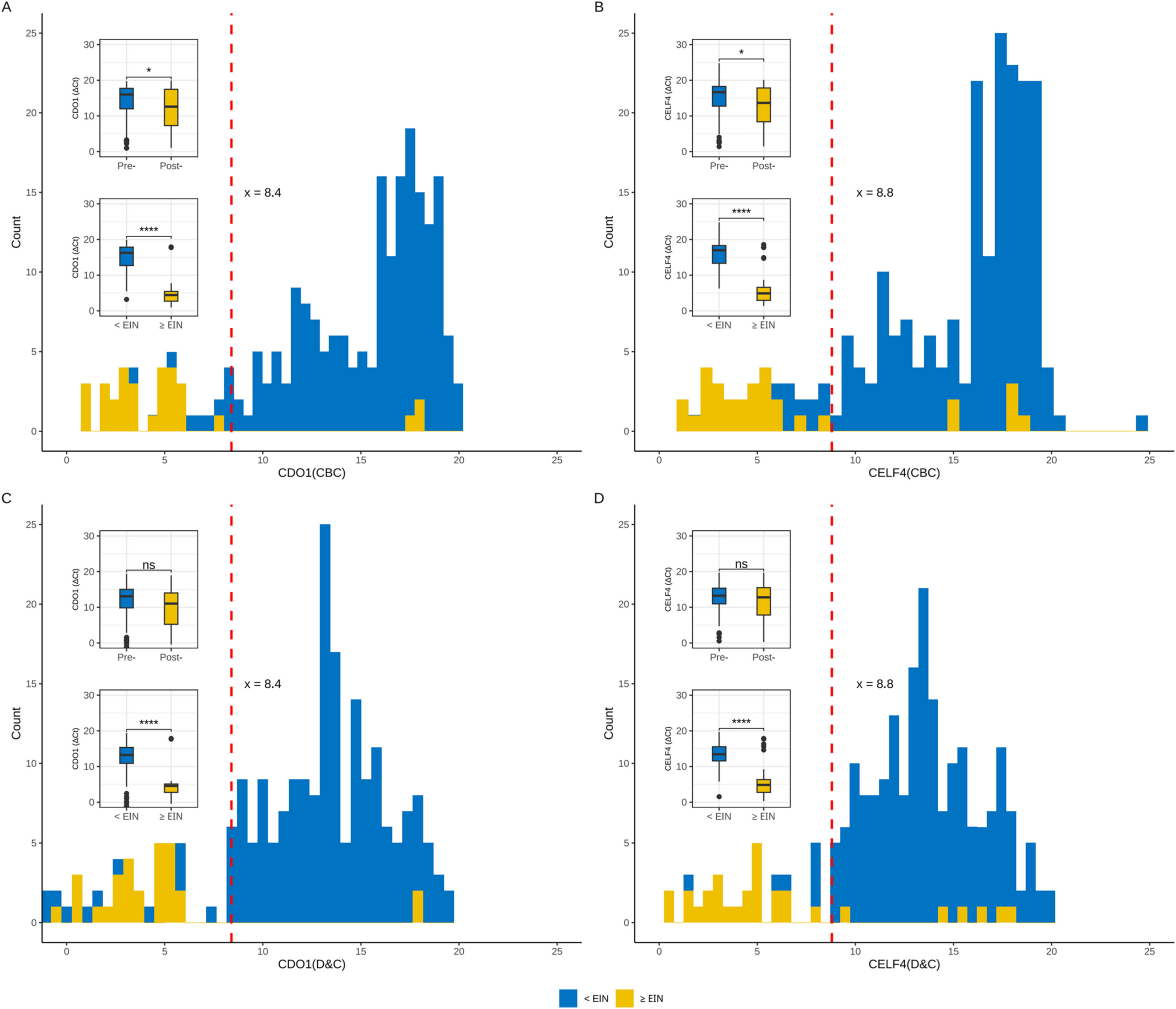
Distribution of ΔCt values for the *CDO1*/*CELF4* methylation test in CBC and D&C samples. This figure presents histograms detailing the distribution of ΔCt values (non-log-transformed) for *CDO1* and *CELF4* methylation results across CBC and D&C samples, stratified by pathological diagnosis ( ≥ EIN; < EIN). Specifically, the ΔCt values of the *CDO1* methylation test from CBC samples are depicted in **(A)**, and those from D&C samples are depicted in **(C)**. Concurrently, the ΔCt values of the *CELF4* methylation test from CBC samples are shown in **(B)**, and those from D&C samples are shown in **(D)**. The red dashed lines indicate the diagnostic cutoffs of 8.4 for *CDO1* and 8.8 for *CELF4*. The inset box plots within each section show comparisons of the ΔCt values based on menopausal status and pathological diagnosis. Statistical significance is denoted as *P < 0.05, ****P < 0.00001, ns, not significant, with lower ΔCt values signifying higher methylation levels.

Briefly, the ΔCt value represents the difference in PCR cycle thresholds between the target methylated gene and the internal reference gene; therefore, a lower ΔCt value indicates a higher level of gene methylation. Among the samples obtained from both CBC and D&C sampling, the ΔCt values for individual methylated *CDO1* and *CELF4* were significantly lower in patients with ≥ EIN than in those with < EIN (*P* < 0.0001 for all comparisons; Figure 2). The distribution of ΔCt values illustrated a clear separation between these two groups, with most samples from the ≥ EIN group having ΔCt values below the respective diagnostic cutoffs of 8.4 and 8.8, whereas the majority of samples from the < EIN group had values above these thresholds. A consistent pattern was observed when comparing EC to non-EC cases, with significantly lower ΔCt values for both methylated *CDO1* and *CELF4* in the EC group across both CBC and D&C specimen types (*P* < 0.0001; Supplementary Figure 1).

Further analysis of menopausal status revealed that, compared with premenopausal women, postmenopausal women in the CBC cohort presented significantly lower ΔCt values for both individually methylated *CDO1* (*P* < 0.05) and *CELF4* (*P* < 0.05) (Figure 2). In D&C samples, however, no significant difference in the ΔCt values of methylated *CDO1* and *CELF4* was found between pre- and postmenopausal women (*P* > 0.05).

### Methylation detection consistency across different sampling methods

**FIGURE 3:**
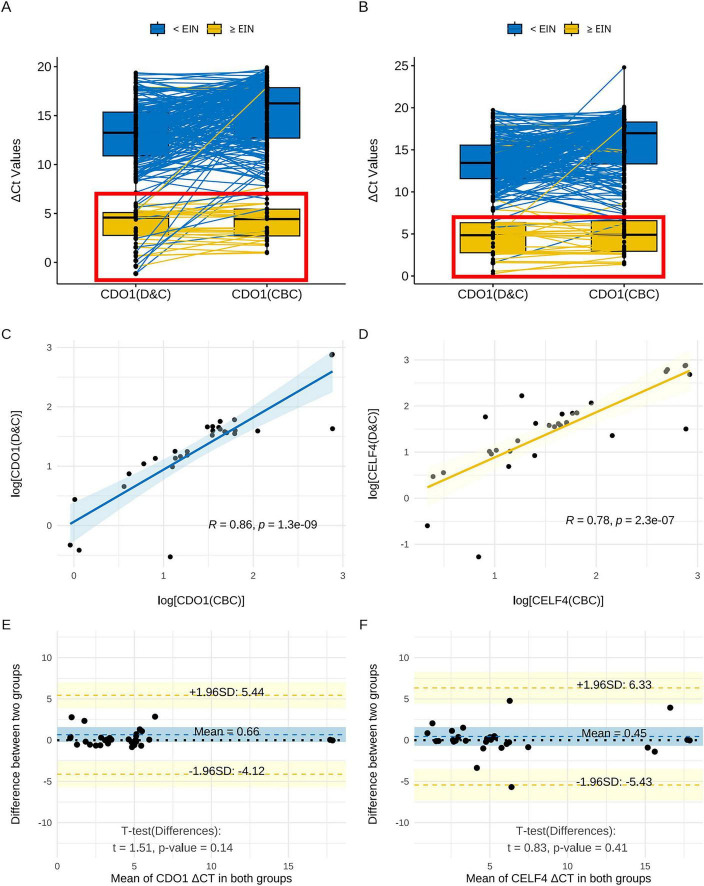
Correlations and agreement of ΔCt values for *CDO1* and *CELF4* methylation between CBC and D&C samples. Paired ΔCt values of *CDO1* **(A)** and *CELF4* **(B)** are shown, with samples from patients with ≥EIN highlighted in yellow and <EIN in blue. Log-transformed ΔCt values demonstrate strong correlations between sampling methods, with Spearman correlation coefficients of 0.86 for CDO1 (**C**, *P* = 1.3 × 10^−9^) and 0.78 for CELF4 (**D**, *P* = 2.3 × 10^−7^). Bland-Altman analyses indicate good agreement between CBC and D&C samples, with mean differences of 0.66 for CDO1 **(E)** and 0.45 for CELF4 **(F)**, and 95% limits of agreement ranging from −4.12 to 5.44 and −5.43 to 6.33, respectively, with no significant systematic bias (*P* = 0.14 for CDO1, *P* = 0.41 for CELF4).

In the two distinct sampling methods, the ΔCt values of the methylated *CDO1/CELF4* gene in ≥ EIN patients are significantly lower than those in < EIN patients; moreover, the ΔCt values of the *CDO1/CELF4* gene in the two types of samples from EIN patients exhibit an identical distribution (Figures 3A,B). A strong positive correlation was identified between the log-transformed ΔCt values obtained from CBC samples and those from D&C samples collected for both individual methylated *CDO1* (R = 0.86, *P* = 1.3e-09) and *CELF4* (*R* = 0.78, *P* = 2.3e-07) (Figures 3C,D). The agreement between the two sampling methods was further assessed via Bland–Altman plots (Figures 3E,F). For the ΔCt values of methylated *CDO1*, the mean difference was 0.66, with 95% limits of agreement ranging from -4.12 to -5.44. Similarly, for the ΔCt values of methylated *CELF4*, the mean difference was 0.45, with 95% limits of agreement ranging from -5.43 to -6.33. For both markers, the differences between the two methods were not statistically significant (indicating no significant systematic bias between the methods; paired *t*-test *P* = 0.14 for *CDO1*; *P* = 0.41 for *CELF4*), indicating a high degree of concordance between the CBC and D&C sampling techniques.

### Diagnostic performance of tests

**FIGURE 4:**
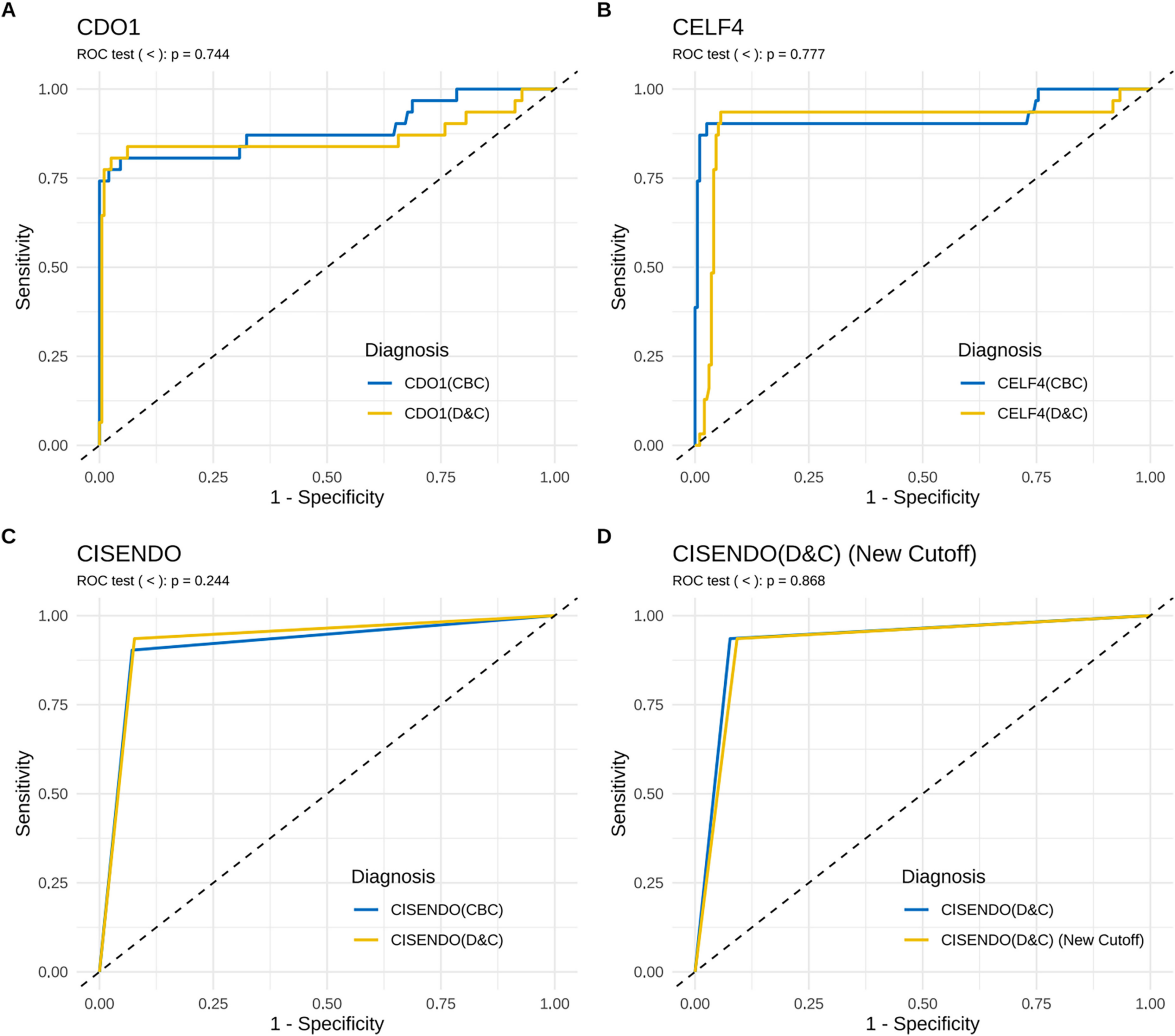
ROC curves for ≥ EIN lesion diagnosis via different methods. Receiver operating characteristic (ROC) curves for various diagnostic methods distinguishing ≥ EIN lesions are presented. **(A)** ROC curves for *CDO1*, comparing CBC and D&C samples (ROC test *p* = 0.744). **(B)** CELF4 curves comparing CBC and D&C samples (ROC test *p* = 0.777). The combined CISENDO curves, which compare CBC and D&C samples (ROC test *p* = 0.244), are shown in **(C)**. Finally, **(D)** contains CISENDO(D&C) curves, which compare its original cutoff with a new cutoff (ROC test *p* = 0.868). The dashed line represents the reference line of no discrimination.

Receiver operating characteristic (ROC) curve analysis was conducted to assess and compare the diagnostic performance of different methods for EIN and EC detection across all participants (Figure 4). The analysis revealed no statistically significant difference in diagnostic accuracy between cervical and uterine samples for methylated *CDO1* (*P* = 0.744) or methylated *CELF4* (*P* = 0.777) alone. When the two markers were combined with the CISENDO test, their diagnostic performance likewise showed no significant difference between the two sampling methods (*P* = 0.244). Furthermore, a comparison between the established CISENDO (D&C) test and a new cutoff of the panel demonstrated that no statistically significant difference was observed between their respective ROC curves (*P* = 0.868). The diagnostic performance of these methylation-based tests was markedly superior to that of traditional markers such as ET (AUC = 0.499) and CA125 (AUC = 0.593) (Table 3). Compared with the CISENDO (CBC) test, the CISENDO (D&C) test achieved a greater AUC of 0.929 (95% CI: 0.881, 0.977), with a sensitivity of 93.5% and a specificity of 92.3% [AUC = 0.916 (0.860, 0.972)].

**TABLE 3:** Sensitivity and specificity for ≥ EIN detection (for all participants).

Methods	AUC (95% CI)	Threshold	Sensitivity	Specificity
Endometrium thickness	0.499 (0.387, 0.611)	9.25 mm	0.548	0.487
CA125	0.593 (0.498, 0.689)	13.25 U/mL	0.677	0.565
Methylated CDO1(CBC)	0.924 (0.845, 1)	7.80	0.903	0.974
Methylated CELF4(CBC)	0.887 (0.801, 0.974)	8.90	0.806	0.954
Methylated CDO1(D&C)	0.906 (0.824, 0.988)	5.99	0.935	0.944
Methylated CELF4(D&C)	0.862 (0.754, 0.969)	9.24	0.839	0.938
CISENDO(CBC)	0.916 (0.86, 0.972)	CDO1: 8.4; CELF4:8.8	0.903	0.928
CISENDO(D&C)	0.929 (0.881, 0.977)	CDO1: 8.4; CELF4:8.8	0.935	0.923
CISENDO(C = BC) (new cutoff)	0.923 (0.868, 0.979)	CDO1: 7.80; CELF4:8.90	0.903	0.944
CISENDO(D&C) (new cutoff)	0.922 (0.873, 0.97)	CDO1: 5.99; CELF4:9.24	0.935	0.908

AUC, area under the curve; CBC, cervical brushing cell; D&C, diagnostic curettage.

A similar analysis was performed specifically for the subgroup of premenopausal women (Supplementary Figure 2). Consistent with the findings in the overall cohort, ROC curve analysis revealed no significant differences in diagnostic accuracy between the cervical and uterine sampling methods for methylated *CDO1* (*P* = 0.997), CELF4 (*P* = 0.960), or the CISENDO test (*P* = 0.800). Similarly, the performance of the new cutoff CISENDO(D&C) was not significantly different from that of the original cutoff CISENDO(D&C) (*P* = 0.868). In this premenopausal cohort, the methylation-based tests again demonstrated superior diagnostic capability compared with ET (AUC = 0.523) and CA125 (AUC = 0.473) (Supplementary Table 4). The methylated *CDO1* (CBC) with the original threshold exhibited the highest AUC of 0.951 (95% CI: 0.862, 1), with 93.8% sensitivity and 99.4% specificity. The CISENDO (CBC) and CISENDO (D&C) tests also performed robustly, with AUCs of 0.943 and 0.933, respectively, and both achieved a sensitivity of 93.8%.

When the analysis focused on the postmenopausal subgroup, the findings remained consistent (Supplementary Figure 3). Diagnostic performance did not differ significantly between sampling methods for individual methylated *CDO1* (*P* = 0.325), *CELF4* (*p* = 0.275), or the CISENDO test (*P* = 0.095) or between the two uterine CISENDO test versions (*P* = 0.500). In this cohort, the methylation markers once again substantially outperformed ET (AUC = 0.615) and performed better than CA125 (AUC = 0.848) did (Supplementary Table 5). The detection capability of CA125 is better in postmenopausal patients. Moreover, methylation detection performed very well in both cohorts.

## Discussion

Our study demonstrated that *CDO1*/*CELF4* methylation level assessment effectively distinguished between benign and EIN/EC (Figure 2). Compared with conventional noninvasive methods for detecting EC, it has superior detection efficacy for ≥ EIN lesions (Table 2). Furthermore, this study provides robust evidence that *CDO1*/*CELF4* methylation analysis of noninvasively collected CBC samples yields results highly concordant with those from invasive D&C samples (Figure 3), and no significant difference in diagnostic performance was observed compared to that achieved using invasively obtained endometrial tissue (Figure 4). Irrespective of menopausal status, the CISENDO^®^ test maintains high accuracy for EIN+ detection (Supplementary Figures 2, 3).

We also found that the CISENDO (CBC) score was slightly better in the premenopausal group (0.943 AUC) than in the postmenopausal group (0.856 AUC), as shown in Supplementary Tables 4, 5. This difference may be attributed to the insufficient number of samples collected via a cervical brush from postmenopausal women. This is likely a consequence of the significant decline in endometrial cell levels due to reduced estrogen and progesterone following menopause (20, 21). In contrast, the CA125 test showed the opposite results. We did not set a specific CA125 threshold, as the majority of participants presented negative results at the 35 U/ml cutoff (Table 2). Among them, 7.69% (12/156) of premenopausal women in the < EIN group and 6.67% (1/15) of postmenopausal women in the ≥ EIN group tested CA125 positive at this cutoff. A large prospective ovarian cancer study in high-risk women suggested defining the CA125 cutoff by menopausal status (50 U/ml for pre- and 35 U/ml for post-) (22). Although CA125 alone is not recommended for EC screening, given its better performance in postmenopausal women, an integrated diagnostic approach combining the highly specific CBC methylation test with serum CA125 assessment could further optimize noninvasive triage for this specific demographic.

The objective of EC screening is to detect atypical hyperplasia or cancerous lesions at their earliest stages, thereby increasing the likelihood of successful treatment and decreasing disease-specific mortality rates (23). Unlike for cervical and breast cancers, a universally accepted standard screening protocol for EC has not yet been established (9). Consequently, the findings of this study underscore the importance of noninvasive cervical brushing cells collected for high-performance *CDO1/CELF4* methylation test in the early diagnosis of EC.

In addition to patient-related impediments for EC detection and diagnosis, such as insufficient awareness of disease risk factors, cultural barriers, and economic constraints (24, 25), current diagnostic methodologies for endometrial lesions are fraught with limitations. Patients typically seek medical intervention due to overt symptoms, including postmenopausal bleeding (PMB). Among postmenopausal women, 90% of those with malignant endometrial lesions present with uterine bleeding; however, only 9% of women with PMB symptoms are ultimately diagnosed with EC (8), often resulting in a considerable number of unnecessary invasive procedures being performed immediately. TVS is a well-tolerated and relatively safe detection tool capable of identifying endometrial abnormalities, such as ET, intrauterine fluid, suspicious polyps, or other atypical features suggestive of an elevated risk of EC (26). An endometrial echo complex of ≤ 5 mm on TVS is deemed adequate for the preliminary assessment of PMB (27). However, TVS heavily depends on operator proficiency and can be confounded by factors such as an axially deviated uterus, adenomyosis, or coexisting fibroids (28). Although TVS has high sensitivity, its low specificity (29) frequently requires supplementary invasive procedures to confirm the findings. Moreover, the optimal ET threshold for women in various menopausal states remains contentious (30), and TVS has poor detection efficacy for type II EC (31, 32). Consequently, alternative diagnostic approaches are imperative to aid clinicians in determining the necessity for further investigation, particularly in postmenopausal women.

Conversely, the application of cervical brash cells, referred to as cytology samples, in evaluating EC risk is currently experiencing advancements. Clinical studies indicate that morphological examination of Pap smears is not an effective tool for the early detection of EC (14); despite its noninvasive nature and convenience, its diagnostic efficacy is unsatisfactory (33). The combination of urine and vaginal swab samples for cytological analysis has a sensitivity of 91.7% and a specificity of 88.8% in patients with unexplained PMB who are suspected of having EC (34). However, its utility is confined to individuals already requiring hysteroscopic evaluation, thus precluding its use as a universal screening method. In Japan, intrauterine sampling for cytological examination has been integrated into routine EC screening protocols; however, it continues to necessitate invasive sampling procedures (35, 36). Intrauterine cytological assessments provide highly accurate diagnostic outcomes for EC, with various sampling devices, such as SAP-1 (37), Tao Brush (38), and Li Brush (38), achieving high sensitivity. Nonetheless, variability exists in sampling and outcomes when different operators employ the same methodology, and endometrial cytology (ECT) does not consistently provide reliable exclusion of EC (39). Additionally, inadequate sampling of ECT remains a challenge that must be addressed to increase the effectiveness of EC screening (40). As a result, supplementary diagnostic evaluations of the local lesion are frequently required subsequent to a benign ECT result (39).

The prevailing diagnostic approach entails a series of TVS examinations, outpatient hysteroscopy, and endometrial biopsies to rule out the presence of malignant conditions; however, this method is inherently flawed (41). Up to 31% of women experience failed outpatient hysteroscopy and endometrial biopsy, often due to technical issues or unbearable discomfort, which necessitates subsequent invasive procedures under general anesthesia (34). Routine, widespread endometrial sampling may be both impractical and potentially superfluous. Nevertheless, individuals at elevated risk for EC necessitate vigilant and regular monitoring (42). Fortunately, the anatomical continuity between the uterine cavity and the lower genital tract facilitates the use of biomaterials derived from cervicovaginal fluid for the noninvasive detection of EC. Compared to invasive D&C, noninvasive CBC sampling offers substantial clinical advantages: it does not require anesthesia, minimizes patient discomfort, and can be easily performed in an outpatient setting. The *CDO1/CELF4* methylation test, a straightforward and easily implementable noninvasive test, can function as an effective triage mechanism for women at risk of EC, offering comfort to the majority of healthy women and thereby optimizing patient management. Crucially, this test demonstrates strong translational potential. Integrating the CBC CDO1/CELF4 methylation test into current primary care screening programs could serve as a highly accurate, point-of-care triage step. This real-world application would effectively reduce the burden of unnecessary invasive procedures, lower healthcare costs, and significantly improve screening compliance for women at risk of EC.

Furthermore, the *CDO1/CELF4* methylation test, acquired through noninvasive sampling techniques and exhibiting remarkable sensitivity and specificity (Table 3), has emerged as a formidable alternative to the prevailing obstacles in the early detection of EC. The diagnostic efficacy of the CISENDO test markedly surpasses the constrained precision of TVS and CA125, providing a substantially more dependable triage instrument (Table 2). Importantly, our results suggest that the CISENDO test can accurately identify both Type I and Type II ECs, irrespective of the sample type utilized (Supplementary Tables 4, 5). This could substantially decrease the number of women subjected to superfluous invasive procedures, thereby concentrating resources on those at greatest risk. Additionally, it circumvents the issue of missed diagnoses. The DNA methylation test has the potential to alleviate the challenge of unnecessary complications, such as pain, infection, bleeding, and vasovagal episodes, which are frequently linked with aberrant TVS findings (43).

Although DNA methylation methods based on CBC specimens have been widely investigated and are permitted in some countries for the auxiliary diagnosis of EC, the anatomical representativeness of CBC samples for determining endometrial lesion status remains questioned by gynecologists. Therefore, the strong correlation and high agreement between CBC and D&C collected samples (Figure 3) are pivotal, thereby validating the use of a simple, noninvasive cervical sample as a reliable surrogate for endometrial tissue. The validation of a noninvasive diagnostic test has significant clinical ramifications. It presents a compelling opportunity to transition from the prevailing reactive, symptom-centric approach to a proactive preventive screening model, particularly for high-risk populations. The utilization of cervical specimens is especially transformative, as it enables the integration of EC screening into standard gynecological practice, complementing existing cervical cancer screening initiatives (7).

This study is strengthened by its paired-sample design and detailed stratification by menopausal status. However, we acknowledge its limitations, including its single-center design and modest sample size. We acknowledge that this limited number of positive cases leads to wider confidence intervals for our diagnostic performance metrics and may reduce the statistical stability of our subgroup analyses, particularly the stratification by menopausal status. Furthermore, the study population consisted of patients referred for hysteroscopy or uterine cavity surgery, representing a high-risk cohort. This spectrum bias means the diagnostic performance metrics may not fully generalize to average-risk population screening or routine primary-care triage settings. Additionally, the optimized ‘new cutoffs’ derived for specific sample types represent exploratory findings. Because they were evaluated on the same dataset used for their derivation without internal cross-validation, their performance may be optimistic and requires rigorous external validation in independent cohorts. Importantly, the absence of a statistically significant difference should not be interpreted as evidence of equivalence, as no formal non-inferiority or equivalence testing was performed. Future large-scale, prospective, multicenter trials are essential to confirm the clinical utility, cost-effectiveness, and generalizability of the CISENDO test in diverse, real-world screening populations.

## Conclusion

*CDO1/CELF4* methylation analysis using cervical brushing samples demonstrates high diagnostic accuracy for detecting EIN and EC, and no significant difference in performance was observed when compared to intrauterine D&C specimens. Although D&C remains the reference diagnostic procedure, cervical brush sampling may represents a reliable, minimally invasive alternative that may improve patient compliance and accessibility. These findings support the potential role of methylation-based triage in symptomatic women. The integration of methylation testing into current triage algorithms may reduce unnecessary invasive procedures while maintaining diagnostic safety. However, external validation in broader populations is required before routine clinical implementation.

## Data Availability

The original data will be available from the corresponding author upon reasonable request.
