# Supplementary material for: Validation of a cervical CDO1/CELF4 methylation test for endometrial cancer: a prospective paired-sample comparison with intrauterine specimen
Source: Front Med (Lausanne). 2026 May 29;13:1690020. doi: 10.3389/fmed.2026.1690020 (PMC13260639; doi:10.3389/fmed.2026.1690020)
Supplement: Supplementary file 1 [file Presentation_1.pdf]

Supplementary Figures

Description of data: This file contains Figures S1-S3 and Tables S1-S5, which provide additional details related to the study.

Figures

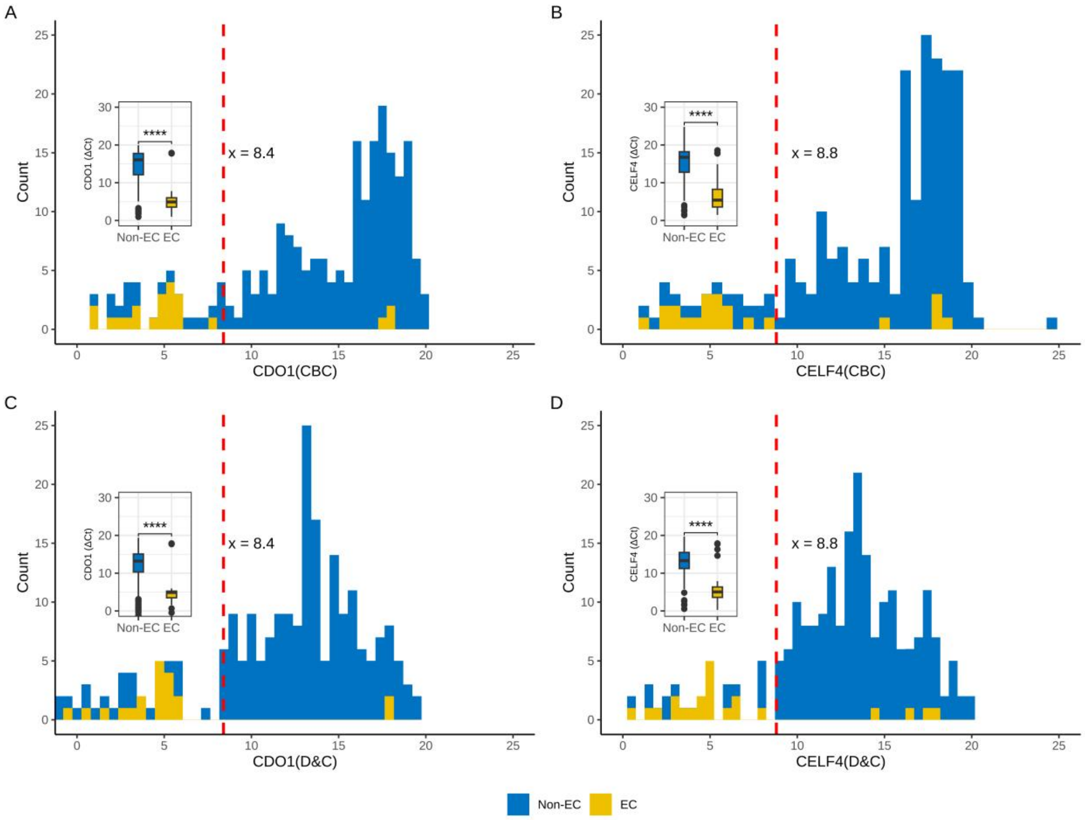

Figure S1: CDO1/CELF4 ΔCt Values Distribution by EC/Non-EC Classification

Histograms detail the distribution of ΔCt values (non-log-transfromed) for CDO1 and CELF4 methylation across cervical brushing cells (CBC) and D&C samples, stratified by pathological diagnosis (EC; Non-EC). For CDO1 ΔCt values, CBC samples are presented in (A), and D&C samples in (C). For CELF4 ΔCt values, CBC samples are in (B), and D&C samples in (D). Red dashed lines indicate the diagnostic cutoffs: 8.4 for CDO1 and 8.8 for CELF4. Inset box plots within each section provide comparisons of ΔCt values based on pathological diagnosis (Non-EC vs. EC). Statistical significance is denoted as:*: $P < 0.05$, ****: $P < 0.0001$, with lower ΔCt values signifying higher methylation levels.

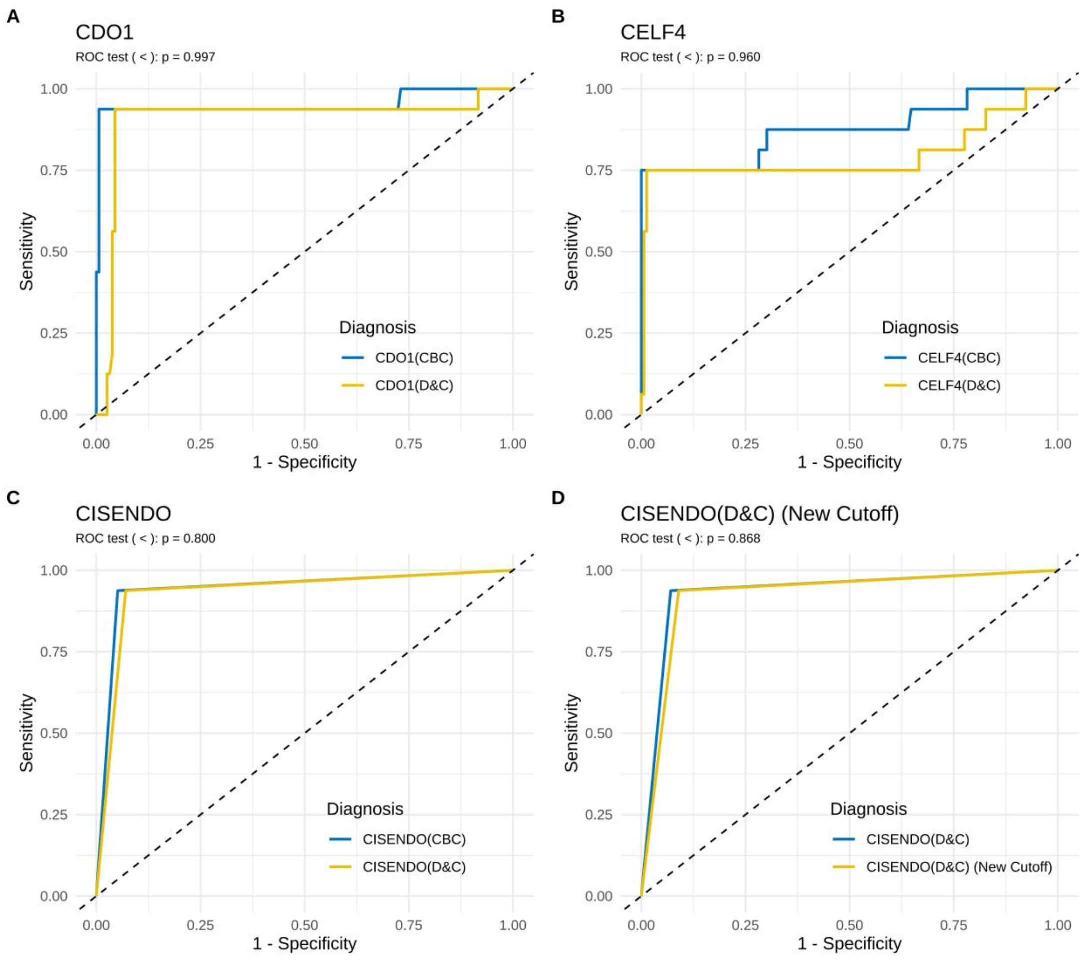

Figure S2: ROC Curves for ≥ EIN Lesion Diagnosis in Premenopausal Women

Receiver Operating Characteristic (ROC) curves for various diagnostic methods distinguishing ≥ EIN lesions in premenopausal women are presented. (A) encompasses ROC curves for CDO1, comparing CBC and D&C samples (ROC test p=0.997). (B) details CELF4 curves, comparing CBC and D&C samples (ROC test p=0.960). The combined CISENDO panel curves, comparing CBC and D&C samples (ROC test p=0.800), are found in (C). Lastly, (D) contains CISENDO(D&C) panel curves, comparing its original cutoff with a new cutoff (ROC test p=0.868). The dashed line represents the reference line of no discrimination.

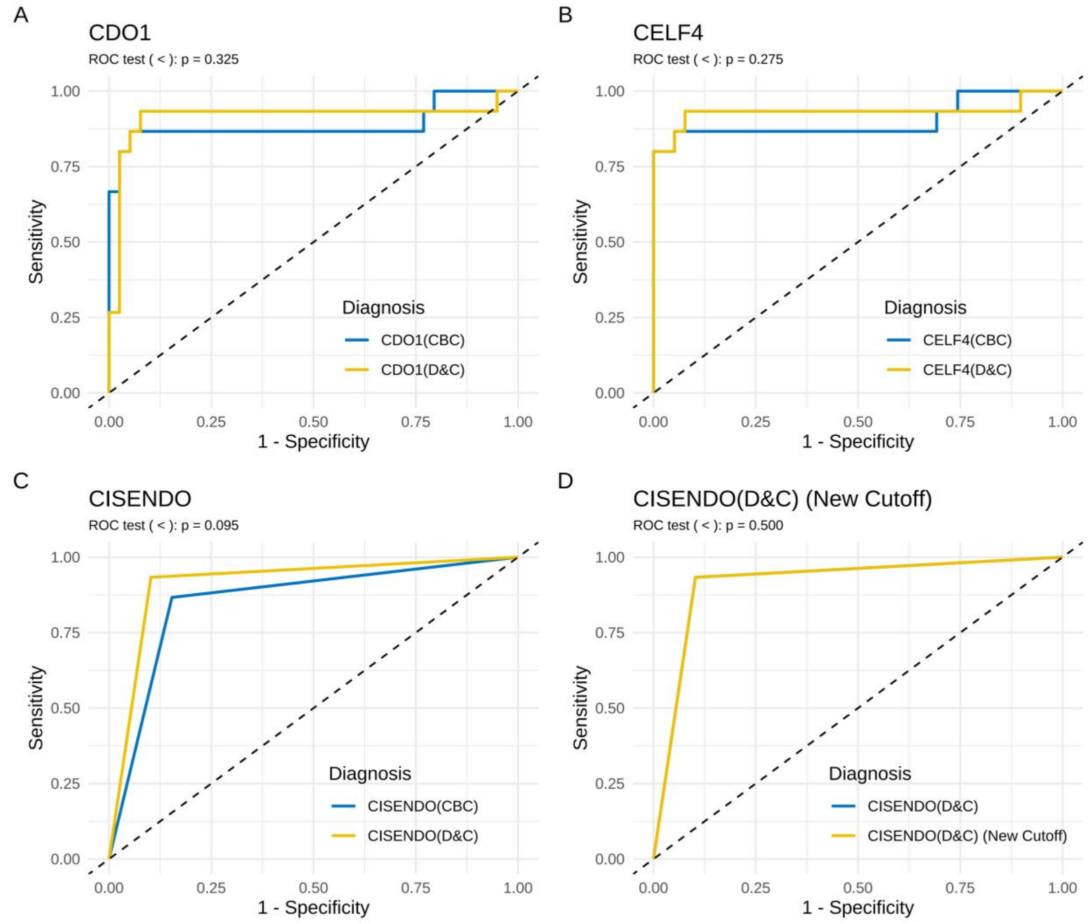

Figure S3: ROC Curves for ≥ EIN Lesion Diagnosis in Postmenopausal Women

Receiver Operating Characteristic (ROC) curves for various diagnostic methods distinguishing ≥ EIN lesions in postmenopausal women are presented. (A) encompasses ROC curves for CDO1, comparing CBC and D&C samples (ROC test P=0.325). (B) details CELF4 curves, comparing CBC and D&C samples (ROC test P=0.275). The combined CISENDO panel curves, comparing CBC and D&C samples (ROC test P=0.095), are found in (C). Lastly, (D) contains CISENDO(D&C) panel curves, comparing its original cutoff with a new cutoff (ROC test P=0.500, the two lines coincide). The dashed line represents the reference line of no discrimination.
